# Rabbit haemorrhagic disease virus *Lagovirus europaeus*/GI.1d strain: genome sequencing, in vivo virus replication kinetics, and viral dose effect

**DOI:** 10.1186/s12917-021-02962-2

**Published:** 2021-07-28

**Authors:** Clément Droillard, Evelyne Lemaitre, Michel Amelot, Yannick Blanchard, Alassane Keita, Nicolas Eterradossi, Ghislaine Le Gall-Reculé

**Affiliations:** 1 Ploufragan-Plouzané-Niort Laboratory, Avian and Rabbit Virology Immunology and Parasitology Unit, French Agency for Food, Environmental and Occupational Health and Safety (ANSES), BP 53, F-22440 Ploufragan, France; 2 Ploufragan-Plouzané-Niort Laboratory, Department for Breeding and Experimentation in Poultry and Rabbits, French Agency for Food, Environmental and Occupational Health and Safety (ANSES), BP 53, F-22440 Ploufragan, France; 3 Ploufragan-Plouzané-Niort Laboratory, Viral Genetics and Biosafety Unit, French Agency for Food Environmental and Occupational Health and Safety (ANSES), BP 53, F-22440 Ploufragan, France

**Keywords:** Lagovirus, RHDV, GI.1d, Replication kinetics, Minimum infective dose, RT-qPCR, *Oryctolagus cuniculus*

## Abstract

**Background:**

Rabbit haemorrhagic disease virus *Lagovirus europaeus*/GI.1d variant (GI.1d/RHDV) was identified in 1990 in France, and until the emergence of the new genotype GI.2, it was the main variant circulating in the country. The early stages of RHDV infection have been described in a few studies of rabbits experimentally infected with earlier strains, but no information was given on the minimum infective dose. We report the genomic and phenotypic characterisation of a GI.1d/RHDV strain collected in 2000 in France (GI.1d/00–21).

**Results:**

We performed in vivo assays in rabbits to study virus replication kinetics in several tissues at the early stage of infection, and to estimate the minimum infective dose. Four tested doses, negligible (10^− 1^ viral genome copies), low (10^4^), high (10^7^) and very high (10^11^) were quantified using a method combining density gradient centrifugation of the viral particles and an RT-qPCR technique developed to quantify genomic RNA (gRNA). The GI.1d/00–21 genome showed the same genomic organisation as other lagoviruses; however, a substitution in the 5′ untranslated region and a change in the potential p23/2C-like helicase cleavage site were observed. We showed that the liver of one of the two rabbits inoculated via the oral route was infected at 16 h post-infection and all tissues at 39 h post-infection. GI.1d/00–21 induced classical RHD signs (depression) and lesions (haemorrhage and splenomegaly). Although infective dose estimation should be interpreted with caution, the minimum infective dose that infected an inoculated rabbit was lower or equal to 10^4^ gRNA copies, whereas between 10^4^ and 10^7^ gRNA copies were required to also induce mortality.

**Conclusions:**

These results provide a better understanding of GI.1d/RHDV infection in rabbits. The genome analysis showed a newly observed mutation in the 5′ untranslated region of a lagovirus, whose role remains unknown. The phenotypic analysis showed that the pathogenicity of GI.1d/00–21 and the replication kinetics in infected organs were close to those reported for the original GI.1 strains, and could not alone explain the observed selective advantage of the GI.1d strains. Determining the minimum dose of viral particles required to cause mortality in rabbits is an important input for in vivo studies.

**Supplementary Information:**

The online version contains supplementary material available at 10.1186/s12917-021-02962-2.

## Background

Rabbit haemorrhagic disease virus (RHDV) is a highly pathogenic virus (genus *Lagovirus*, family *Caliciviridae*) that causes rabbit haemorrhagic disease (RHD) in wild and domestic European rabbits (*Oryctolagus cuniculus)*. The *Lagovirus* genus also comprises the pathogenic European brown hare syndrome virus (EBHSV), first detected in Sweden in 1980 [1]. RHD was initially reported in 1984 in China, and subsequently in many countries throughout the world [2]. RHD is characterised by acute fulminant hepatitis, splenomegaly, haemorrhage and congestion in several organs such as the trachea, lungs, heart and kidneys, mainly associated with massive disseminated intravascular coagulation [2]. Infected rabbits over 8 weeks of age succumb within 12 to 36 h post infection (hpi), with case fatality rates between 70 and 90% [3]. In 2010, a new genotype designated RHDV2 (or RHDVb) was first detected in France in both RHDV-vaccinated and unvaccinated domestic rabbits, together with wild rabbits [4]. RHDV2 causes a disease similar to that due to RHDV, except that the course of the disease and case fatality rates are more variable, and that kittens under 4 weeks old and various hare species can also be infected [5].

RHDV has a polyadenylated positive-sense single-stranded RNA genome of about 7.5 kb, comprising two open reading frames (ORFs) [6]. ORF1 codes for a polyprotein that is cleaved by the virus-encoded trypsin-like cysteine protease into eight proteins: seven non-structural proteins (p16, p23, helicase, p29, VPg, Protease, and RdRp) and the major structural protein corresponding to the capsid protein (VP60). ORF2 codes for a minor structural protein, VP10. A subgenomic polyadenylated RNA (sgRNA) of about 2.2 kb and colinear to the 3′ end of the gRNA is also produced during replication. It comprises two ORFs that code for the VP60 and the VP10 proteins, respectively [7]. Recombination events have frequently been observed in lagovirus genomes, with a recurrent breakpoint at the boundary between non-structural and structural encoding genes (between RdRp and VP60). Recombination events were described as playing a major role in the evolutionary mechanisms of these viruses [8].

A recent proposal for a unified classification system for lagoviruses defined a single species of lagovirus called *Lagovirus europaeus* [9]. Based on the complete capsid protein (VP60) gene sequences, the species was divided into two genogroups GI and GII. GI contains four genotypes related to RHDV (GI.1 to GI.4). GI.1 (RHDV) and GI.2 (RHDV2) are composed of pathogenic viruses for one or several leporid species, whereas GI.3 and GI.4 are composed of benign viruses infecting rabbits. GI.1 is subdivided into several variants G1.1a– GI.1d (previously classified into G1-G5 or classical RHDV and G6 or RHDVa). After the emergence of GI.1 in the 1980s, two variants, GI.1b and GI.1c, co-circulated in Asia and Europe. In Europe, except in the Iberian Peninsula where GI.1b persisted, these two variants were replaced by a new variant GI.1d [10, 11]. After the emergence of GI.2, GI.1d strains became a minority but continued to be occasionally identified in France [12]. GII contains pathogenic (EBHSV) and non-pathogenic viruses (hare calicivirus (HaCV)) related to EBHSV [9]. Currently, no cell culture allows lagovirus replication and viral infection can only be studied in vivo. In natural GI.1 infection, the oral and nasal routes are both considered major for virus transmission [13]. The main target organs of the virus are the liver, the spleen and the lungs, but hepatocytes were shown to be the primary target cells for viral replication [14]. The replication kinetics and the minimum infectious dose have been poorly studied. The early stages of GI.1 lagovirus infection have been described in a few studies of rabbits experimentally infected with earlier GI.Ib and GI.Ic strains. These studies monitored the time course of virus distribution, from 2 hpi [15], 6 hpi [16] or 12 hpi [14, 17] onwards, in several organs of rabbits infected by the nasal, intra-muscular or subcutaneous routes, and by using RT-PCR, Western blot or immunohistochemistry to detect the virus in the infected organs. However, no information was given on the minimum dose of virus particles that can cause infection in inoculated rabbits, and notably by a natural route. In addition, no study of pathogenicity and replication kinetics is yet available for GI.1d strains, although these strains have replaced the earlier GI.1b and GI.1c strains in several European countries, which could suggest a selective advantage of the former over the two latter variant GI.1 viruses.

In this study, we obtained the full-length genome sequence of a GI.1d strain collected in France in 2000, and characterised its genomic organisation. Additionally, we characterised its phenotype and we assessed its replication kinetics in 10-week-old specific-pathogen-free New Zealand White rabbits*.* We also inoculated by the oral route different virus doses to assess the minimum infective dose of this strain to better understand GI.1d infection in rabbits.

## Results

### Genome characterisation of the GI.1d/00–21 strain

**Fig. 1:**
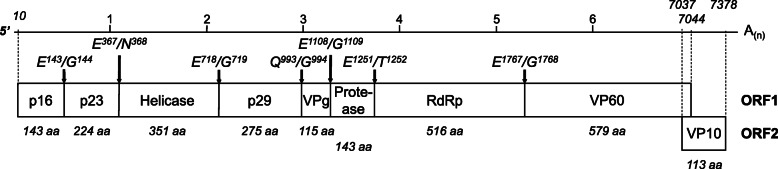
Genome organisation of the GI.1d/00–21 strain. The viral genome is 7433 nucleotides in length, excluding the poly(A) tail. The genomic RNA is organised into two open reading frames (ORFs). ORF1 is 7044 nt long and encodes a 2346 amino-acid (aa)-long polyprotein (p16, p23, Helicase, p29, VPg, Protease, RdRp, VP60), and ORF2 is 342 nt long and encodes a 113-aa-long protein (VP10). The expected cleavage sites are illustrated at the top of the genome using black arrows

The GI.1d/00–21 genome sequence (accession number MH190418) was 7437 nucleotide (nts) in length, excluding the poly(A) tail. When compared with the other GI.1 genomes, a T/C substitution was detected at position 8 of the 5′ untranslated region (UTR). The genome organisation and the expected cleavage sites previously described for the GI.1 polyprotein proteolytic processing [7] are illustrated in Fig. 1. ORF1 has the same order of non-structural proteins (p16, p23, 2C-like helicase, p29, VPg, 3-C like protease and RNA-dependent RNA polymerase RdRp) and major structural protein (capsid protein VP60) obtained after the proteolytic processing of the polyprotein as that of the other lagoviruses. However, a change in the potential p23/2C-like helicase cleavage site (dipeptide E^367^/N^368^ instead of E/D) was observed. ORF2 codes for the minor structural protein VP10. No recombination events with known lagoviruses were detected (data not shown).

**Fig. 2:**
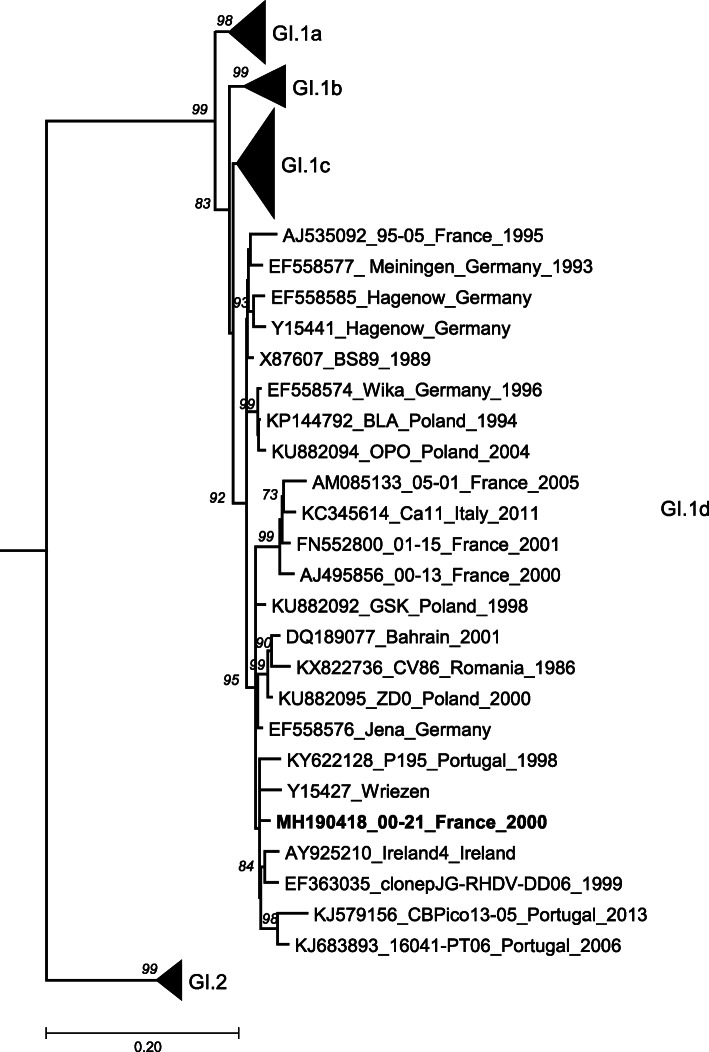
Maximum likelihood (ML) phylogenetic tree of lagovirus GI.1 and GI.2 VP60 gene nucleotide sequences (*n* = 121). The tree was rooted using a GII.2/EBHSV VP60 sequence (accession number AM933650). The sequences were selected with Jalview software (redundancy threshold of 98%) from the 631 complete lagovirus GI.1 and GI.2 VP60 gene sequences available in the GenBank database at the time of analysis. GI genotypes and variants are represented as collapsed in order to reduce the tree size and highlight the newly identified sequence. The sample newly sequenced in this study is shown in bold, and significant (greater than 70) bootstrap values for 1000 replicates are shown in italics after each node. Scale bar indicates nucleotide substitutions per site

Results of the phylogenetic analyses based on the VP60 gene nucleotide sequences of 121 selected GI.1 and GI.2 lagoviruses are shown in Fig. 2. The results revealed that the strain belonged to the GI.1d variant according to the nomenclature proposed by Le Pendu et al. [9].

### In vivo replication kinetics, clinical signs and case fatality rates

**Fig. 3:**
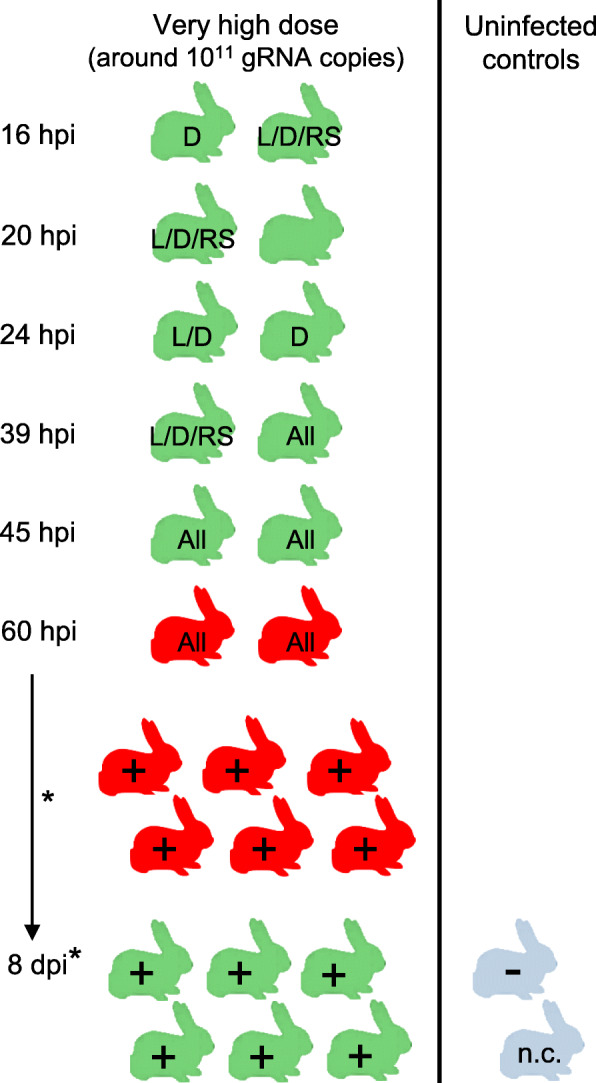
Experimental design to study replication kinetics, clinical signs and case fatality rates in GI.1d/00–21-infected rabbits. Twenty-four rabbits (visualised in green or in red) were inoculated with a very high infective dose (around 10^11^ gRNA copies/rabbit) and two rabbits were euthanised at 16, 20, 24, 39 and 45 h post-infection (hpi), respectively. The assay was ended at 8 days post-infection (dpi). Rabbit in green: euthanised rabbit without RHD sign, rabbit in red: rabbit that died of RHD or was euthanised after reaching humane endpoints. Rabbit in light blue: uninfected control rabbit. * Blood and liver samples were collected from euthanised rabbits and only liver samples from animals found dead. RT-qPCR-positive samples (Table 1): L: liver, D: duodenum, RS: rectal swab, All: all the collected samples. +: detection of viral genome by RT-qPCR in the liver; −: absence of detection of viral genome by RT-qPCR in the liver; n.c.: sample not collected

Replication kinetics in GI.1d/00–21 experimentally inoculated rabbits via the oral route with a very high infective dose (around 10^11^ gRNA copies/rabbit) were studied by quantifying viral genomes collected from different organs of two rabbits euthanised at 16, 20, 24, 39 and 45 hpi, respectively, as well as from the two first rabbits that died of the disease at 60 hpi (Fig. 3). No RHD signs were observed in infected rabbits during the first 45 hpi, and no macroscopic lesions were observed at necropsy. Viral genomes were detected by RT-qPCR in the liver, duodenum and the rectal swab supernatant at 16 hpi, consistently in these samples from 39 hpi, and consistently in other tissues from 45 hpi. In detail, viral genomes were detected in the liver, duodenum and rectal swab supernatant from one rabbit euthanised at 16 hpi and one rabbit euthanised at 20 hpi. Only the duodenum sample from the second rabbit euthanised at 16 hpi was found to be positive by RT-qPCR, and no viral genomes were detected in the tissue samples from the other rabbit euthanized at 20 hpi. At 24 hpi, viral genome was detected in the duodenum sample from one rabbit, and both duodenum and liver samples from the other rabbit. At 39 hpi, viral genome was detected in the liver, duodenum and rectal swab supernatant from one rabbit, and in all the tissues from the other rabbit. All subsequent tissue samples were found to be positive by RT-qPCR (Fig. 3). The numbers of log_10_ viral genome copies per μL RNA are presented in Table 1.

**Table 1:** Viral copies of GI.1d/00–21 per μL RNA from experimentally infected rabbits (around 10^11^ gRNA copies/rabbit)

Sampling time	Collected tissues							
	Liver	Duodenum	Rectal swab	Thymus	Lung	Spleen	Kidney	Faeces
16 hpi	neg.	4,31	neg.	neg.	neg.	neg.	neg.	neg.
	2,50	4,72	2,20	neg.	neg.	neg.	neg.	neg.
20 hpi	2,75	2,72	2,56	neg.	neg.	neg.	neg.	neg.
	neg.	neg.	neg.	neg.	neg.	neg.	neg.	neg.
24 hpi	3,77	2,71	neg.	neg.	neg.	neg.	neg.	neg.
	neg.	2,20	neg.	neg.	neg.	neg.	neg.	neg.
39 hpi	3,65	3,40	3,25	neg.	neg.	neg.	neg.	neg.
	7,32	3,91	3,36	5,38	6,11	2,51	4,95	3,72
45 hpi	5,85	3,70	2,91	4,97	4,15	4,11	4,15	2,26
	7,08	4,15	3,93	5,00	5,59	3,11	5,57	3,18
60 hpi^a^	7,92	4,26	4,08	6,28	5,18	3,73	6,18	4,04
	7,34	4,32	3,95	5,91	6,90	6,63	6,64	3,04
3 dpi^b^	8,10	n.c.	n.c.	n.c.	n.c.	n.c.	n.c.	n.c.
	8,10	n.c.	n.c.	n.c.	n.c.	n.c.	n.c.	n.c.
4 dpi^c^	8,26	n.c.	n.c.	n.c.	n.c.	n.c.	n.c.	n.c.
	7,92	n.c.	n.c.	n.c.	n.c.	n.c.	n.c.	n.c.
4,5 dpi^c^	8,23	n.c.	n.c.	n.c.	n.c.	n.c.	n.c.	n.c.
5 dpi^c^	7,72	n.c.	n.c.	n.c.	n.c.	n.c.	n.c.	n.c.
8 dpi^d^	4,89	n.c.	n.c.	n.c.	n.c.	n.c.	n.c.	n.c.
	2,86	n.c.	n.c.	n.c.	n.c.	n.c.	n.c.	n.c.
	3,90	n.c.	n.c.	n.c.	n.c.	n.c.	n.c.	n.c.
	4,08	n.c.	n.c.	n.c.	n.c.	n.c.	n.c.	n.c.
	3,23	n.c.	n.c.	n.c.	n.c.	n.c.	n.c.	n.c.
	5,04	n.c.	n.c.	n.c.	n.c.	n.c.	n.c.	n.c.
Uninfected control rabbits	neg.	neg.	neg.	neg.	neg.	neg.	neg.	neg.
8 dpi	n.c.	n.c.	n.c.	n.c.	n.c.	n.c.	n.c.	n.c.

The gRNA copies were calculated from RT-qPCR analyses (log_10_); *hpi* hour post-infection, *dpi* day post-infection, *neg* negative RT-qPCR result, *n.c.* sample not collected, ^a^ the first died or euthanised after reaching humane endpoints rabbits were reported at 60 hpi, ^b^ rabbits euthanised after reaching humane endpoints, ^c^ rabbits that died of the disease, ^d^ surviving infected rabbits euthanised at the end of the trial (8 dpi)

**Fig. 4:**
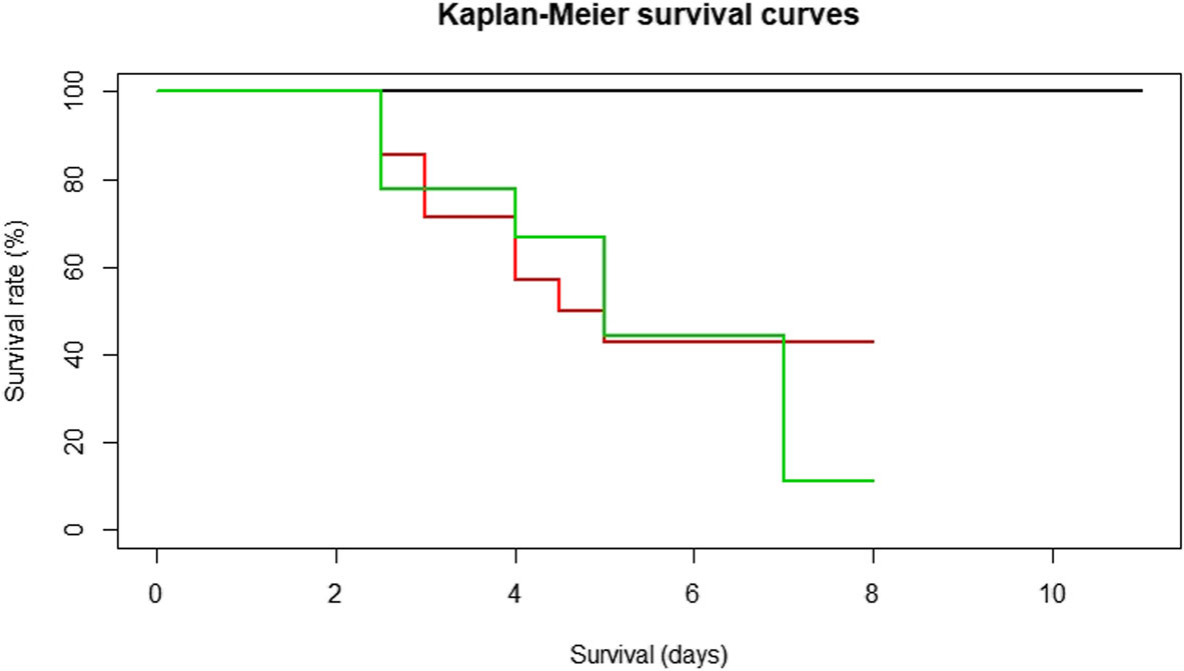
Survival analysis of rabbits challenged with GI.1d/00–21 at four doses of genomic RNA copies. The 10^− 1^ and 10^4^ gRNA copy doses (9 inoculated rabbits) are illustrated in black, the 10^7^ gRNA copies dose (9 inoculated rabbits) in green, and the very high dose (around 10^11^ gRNA copies/rabbit; 14 inoculated rabbits) in red. The 10 rabbits inoculated with the very high dose that were euthanised for kinetics analysis were not included in this analysis. Surviving rabbits were euthanised at eight days post infection (dpi) for the very high dose and at 11 dpi for the 10^− 1^ and 10^4^ gRNA copy doses. The survival analysis for the 10^7^ gRNA copy dose was stopped at 8 dpi when the non-inoculated in-contact rabbit died

Characterisation of the RHD signs and case fatality rates was performed from the 14 rabbits surviving beyond 45 hpi (Fig. 3). The first rabbits that died from RHD were reported at 60 hpi (2 rabbits) and no RHD signs were observed during the previous visit to check for signs of terminal disease (49 hpi). Eight rabbits died of the disease or were euthanised after reaching humane endpoints. They showed RHD signs and lesions at necropsy typical of RHD such as haemorrhagic liver, lungs and trachea, as well as splenomegaly [2]. The six surviving rabbits euthanised at the end of the trial at 8 days post-infection (dpi) showed no macroscopic lesions. The case fatality rate was 57% (8/14). The survival curve for rabbits inoculated with the very high dose (around 10^11^ gRNA copies) is illustrated in red in Fig. 4. Viral genome was detected in the 14 liver samples by RT-qPCR but in a much lower amount in surviving rabbits at the end of the trial (Table 1).

Sera collected from rabbits euthanised for the replication kinetics study until 45 hpi and from two rabbits euthanised at 3 dpi following signs of terminal disease were analysed. Only the serum of one rabbit euthanised at 3 dpi was found to be positive by RHDV-ELISA showing the beginning of seroconversion.

No RHD signs nor mortality were observed in the two uninfected controls. At the end of the trial (8 dpi), no macroscopic lesions were observed at necropsy, and the tissue samples collected from the sampled rabbit remained free of GI.1d/00–21 RNA (Table 1). Blood samples from both rabbits were found to be negative by RHDV-ELISA.

### In vivo viral genome dose effect

**Fig. 5:**
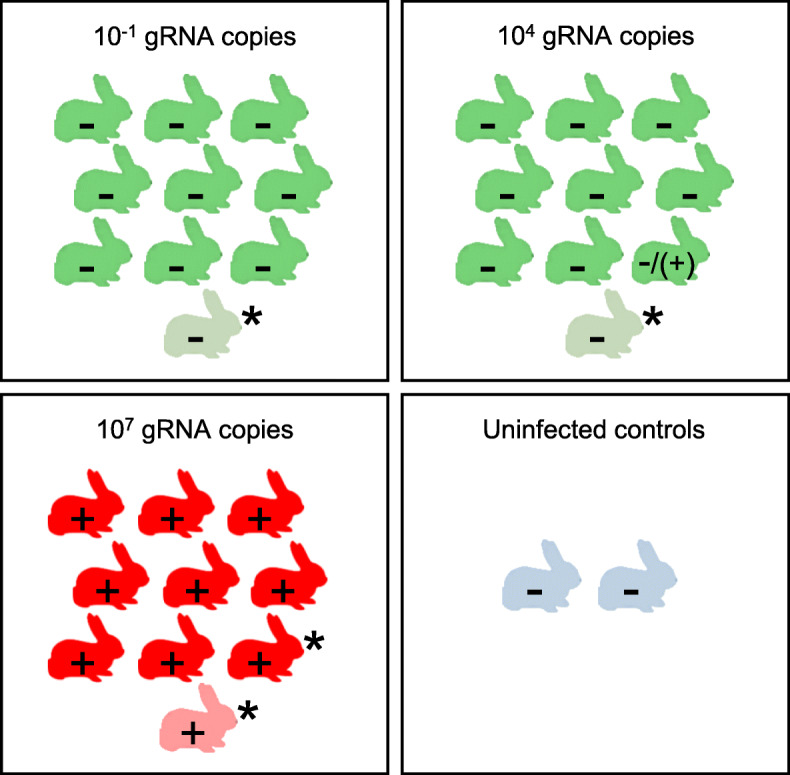
Experimental design to study the viral dose effect in GI.1d/00–21-infected rabbits. Nine rabbits per cell (visualised in green or in red) were orally inoculated with a negligible infective dose (10^− 1^ gRNA copies/rabbit), a low infective dose (10^4^ gRNA copies/rabbit) or a high infective dose (10^7^ gRNA copies/rabbit), respectively, the tenth rabbit (visualised in light green or in pink) was not inoculated (in-contact rabbit). Rabbit in light blue: uninfected control rabbit. +: detection of viral genome by RT-qPCR in the liver; −: absence of detection of viral genome by RT-qPCR in the liver; (+): detection of viral genome by classical RT-PCR in the liver; * not included in calculation of the mortality rate

The minimum infective dose was estimated by orally inoculating 9/10 rabbits per group with 10^− 1^ (negative control dose), 10^4^ (low dose) or 10^7^ (high dose) gRNA copies. The tenth rabbit was not inoculated (in-contact rabbit) (Fig. 5). The case fatality rate of the rabbits inoculated with the very high dose and that died naturally from RHD during the characterization assay of the GI.1d/00–21 phenotype was included in this study.

No mortality was observed in the nine rabbits inoculated with 10^− 1^ or 10^4^ viral genome equivalents (0/9) and in the in-contact rabbit of both groups (Fig. 5). The survival curves are illustrated in black in Fig. 4. There was a statistically significant difference in the log-rank test between rabbits inoculated with 10^− 1^ or 10^4^ gRNA copies survival curves and that of the rabbit inoculated with the very high dose (around 10^11^ gRNA copies) (log-rank test, *p* ≤ 0.01). No macroscopic lesions were observed at necropsy and the 20/20 liver samples were found to be negative by RT-qPCR. However, a liver sample was found to be weakly positive by classical RT-PCR in 1/9 rabbit inoculated with 10^4^ gRNA copies (Fig. 5). No antibodies to RHDV antigen were detected in the sera collected at the beginning and at the end of the trial (11 dpi) with the RHDV-ELISA kit used.

Mortality was observed in 9/9 rabbits inoculated with the dose estimated at 10^7^ gRNA copies (including two rabbits that were euthanised at 5 and 7 dpi after reaching humane endpoints), and in the in-contact rabbit at 8 dpi (Fig. 5). Only 8/9 inoculated rabbits were included in the calculation of the case fatality rate because the contact rabbit was the second last rabbit to die of RHD and we therefore considered that the last inoculated rabbit could have died by horizontal transmission of RHDV. The case fatality rate was 89% (8/9). The survival curve is illustrated in green in Fig. 4. A statistically significant difference was shown between the survival curve of the rabbits inoculated with the 10^7^ dose and those of the rabbits inoculated with the 10^− 1^ and 10^4^ doses (log-rank test, *p* ≤ 0.0001), but not with that of the rabbits inoculated with the 10^11^ dose (log-rank test, *p* = 0.43). All rabbits (10/10) exhibited typical lesions due to RHDV infection and genomic RNA was detected by RT-qPCR in all liver samples (from 6.39 log_10_ to 8.38 log_10_ gRNA copies/μL RNA). Blood samples collected at the beginning of the trial and those collected from the two euthanised rabbits at 5 and 7 dpi were found to be negative with the RHDV-ELISA kit used.

## Discussion

The 00–21 strain belongs to the GI.1d variant group according to the proposal for a unified classification system for lagoviruses [9]. Complete or almost complete GI.1d genome sequences (without complete UTR sequences) from Bahrain in 2001 and from European countries collected until 2004 have been already obtained [11, 18, 19], but not from France where this variant has circulated since 1990 [10, 12]. The present study characterised the complete genome sequence of a GI.1d strain collected in France in 2000. GI.1d/00–21 genome organisation is typical of that of a lagovirus. Nevertheless, the potential p23/2C-like helicase cleavage site (dipeptide E^367^/N^368^) appears unique since that of other lagoviruses is E/D or E/E at this position. However, an E/N cleavage site has been identified at the junction of 3C-like protease and RdRp for GII.1/EBHSV [20, 21] and GII.2/HaCV [22], suggesting that the efficacy of p23/2C-like helicase cleavage should not be affected. Although not all GI.1 5′ UTR sequences are available, the T/C nucleotide substitution observed at position 8 of the 5′ UTR is not shared with any other. However, the first six nucleotides conserved among all lagovirus sequences are also conserved for GI.1d/00–21. The 5′ UTR was described to interact with host factors in other caliciviruses [23], but the role of the lagovirus 5′ UTR is unknown.

Determining the viral replication kinetics in tissues in the early stages of infection as well as the minimum infective viral dose that can initiate the infection is important to understand the pathogenesis of a viral infection. Titration of infectious viral particles is usually performed using cell culture, even though the infectivity of the virus may be different from the infectivity in the host [24]. As no efficient in vitro replication system has been established for RHDV, cell culture titration of infectious lagovirus particles is not possible. Susceptible rabbit inoculations therefore remain the appropriate way for measuring viral infectivity in an inoculum expressed as a median lethal dose (LD_50_) but this method should be avoided as much as possible for welfare considerations [3]. The other method that can be used for RHDV titration is the haemagglutination test (HA). This method is based on the ability of RHDV to agglutinate human erythrocytes (especially type O). However, HA may give false-negative results in presence of HA-negative RHDV variants or of degraded virus particles (smooth particles) that often exist in the organs of rabbits with subacute or chronic forms of RHD [3]. In addition, some RHDV may exhibit temperature-dependent differences in haemagglutinating activity [3]. Development of quantitative real-time RT-PCR methods for the detection and the quantification of viral RNA provides an alternative method for non-cultivable virus enumeration [25], being aware that the titre of a virus expressed in terms of genome copies does not correspond to the actual number of infectious virus particles involved in an in vivo infection. Therefore, to better estimate the infectious viral load of the inocula used for the in vivo assays, we carried out a viral purification step beforehand. Importantly, the direct quantification of viral RNA from infected liver homogenates may introduce a bias by also quantifying the genomes of non-infectious particles. With this in mind, we performed centrifugation through an iodixanol gradient using the method developed by Teixeira et al. [26] for RHDV. This purification method was shown to offer a high degree of purity without any impact on virus viability [26]. However, the potential loss of infectivity of the viral suspension through the process has not been studied. In addition, the RT-qPCR developed in our study targeted the non-structural part of the genome, in contrast to the RT-qPCR described in the literature that targeted the VP60 gene [27–29]. Since the VP60 gene is located in both genomic and subgenomic RNA, targeting this gene may allow for better RT-qPCR sensitivity in particular for diagnostic purposes, but may overestimate the infectious viral load. In contrast, our PCR targeted a portion of the genome that is present in the genomic RNA only, thus theoretically achieving a better correlation between the number of full-length viral genomes detected and the number of infectious particles present in the sample. This approach makes it possible to quantify, as accurately as possible, the number of viral particles inoculated or contained in tissue samples of infected rabbits, being mindful that this quantification cannot be extrapolated to a precise infective dose.

In this study, the GI.1d/00–21 strain induced typical RHD signs and lesions in rabbits inoculated with 10^7^ gRNA copies (high dose) and the very high dose (around 10^11^ gRNA copies). GI.1d/00–21 genomes were detected from the first two rabbits sampled at 16 hpi, in small amounts in the liver and the rectal swab supernatant for one rabbit (2.50 and 2.20 log_10_ viral genome copies/μL RNA, respectively), and in larger amounts in the duodenum for both (4.31 and 4.72 log_10_ viral genome copies/μL RNA). The detection of viral genome in the duodenum and the rectal swabs supernatant was not surprising after an inoculation via the oral route. However, we showed that the virus quickly spread to the liver and was present at 16 hpi. This observation is consistent with studies on RHD performed by Guittré et al. [15] and Shien et al. [16] who detected RHDV RNA by RT-PCR in the liver of infected rabbits euthanised at 18 hpi, and by Prieto et al. [14] who detected VP60 proteins within a few hepatocytes of the first rabbit sampled at 12 hpi. Discrepantly, Alonso et al. [17] were not able to detect VP60 in infected rabbits euthanised at 12 and 24 hpi, perhaps due to the use of different assay conditions [14]. Nevertheless, we did not detect GI.1d/00–21 genomes in the spleens of rabbits at 16 hpi, unlike Guittré et al. [15] and Shien et al. [16] at 18 hpi. At 20 and 24 hpi, when detected, the viral genome load in duodenum samples had decreased, in contrast with the viral load in liver samples. However, at 20 hpi, these results were obtained only from a single rabbit. Samples collected from the other rabbit at 20 hpi were negative for viral RNA, suggesting natural resistance, inoculation problems or too short time to detect viral RNA in other tissues. The decrease in viral genome load in the duodenum could be explained by the elimination of the inoculum in the rabbit digestive tract, whereas the larger amounts of viral RNA in the liver could indicate the beginning of viral replication in this organ. Indeed, previous studies on RHD showed that the liver was the target organ of pathogenic strains, with notably the histological observation of hepatitis lesions starting at 30 hpi [30]. From 39 hpi, all tissue samples were found to be positive by RT-qPCR for all rabbits, except for the second rabbit euthanised at 39 hpi, for which only the liver, duodenum and rectal swab supernatant were positive for viral RNA. Guittré et al. [15] also reported this individual variability at 36 hpi and no longer at 48 hpi. In our study, the first rabbits died spontaneously at 60 hpi, and between 24 and 45 hpi, the virus spread to all harvested tissues without causing macroscopic lesions in organs, in agreement with the findings reported by Plassiart et al. [30]. As previously shown for the liver [15, 16], our study also demonstrated that the viral genome load increased in all organs collected from infected rabbits.

Regarding the estimate of the minimum dose of infectious GI.1d/00–21 virus inducing mortality, no mortality was observed at 10^− 1^ gRNA copies (negative control dose) and 10^4^ gRNA copies (low dose) inoculated to rabbits. These results were expected for the negligible dose but, in principle, less so for the 10^4^ gRNA copies dose. Although no data are available for GI.1 viruses, the minimum infective dose for noroviruses, a calicivirus infecting humans, has been estimated to be between 1 and 100 viral particles [31]. However, genomic RNA was weakly detected by classical RT-PCR in the liver of a rabbit inoculated with the 10^4^ gRNA copies dose, revealing that the virus replicated in this organ without causing mortality. It is surprising that no seroconversion was detected in the rabbits at 11 dpi, especially in the RT-PCR-positive rabbit. This could be explained by a lack of sensitivity of the ELISA test used and/or the short time for seroconversion to start following the oral inoculation of a low viral dose. Oral infection has indeed been shown to extend the survival time of RHDV-infected rabbits (20–22 h) compared to the intramuscular or intradermal routes [32]. When inoculating 10^7^ genome copies, an 89% case fatality rate was obtained. This rate has generally been described for RHD [3]. Finally, the very high dose (around 10^11^ gRNA copies) caused only a 57% case fatality rate. This result could be explained by the insufficient duration of the experiment, during which all rabbits were euthanised at 8 dpi instead of 11 dpi in the other trials. However, no statistically significant differences were observed between the corresponding survival curve and that of the high dose (10^7^ gRNA copies). Therefore, the results of the in vivo viral genome dose assay showed that GI.1d/00–21 needed 10^4^ gRNA copies or less to infect 1/9 susceptible rabbits, and between 10^4^ and 10^7^ gRNA copies to cause mortality.

## Conclusions

The GI.1d/00–21 genome sequence showed specific characteristics with a mutation in the 5′ UTR and a potential p23/2C-like helicase cleavage site E^367^/N^368^. The role of these specific genomic characteristics is still unknown. In vivo assays in normalised conditions were required to estimate the pathogenicity and to better understand the early stages of GI.1d infection in rabbits. The pathogenicity of GI.1d/00–21 and its replication kinetics in the organs of infected rabbits were very close to those published for the original GI.1b and GI.1c strains, and cannot alone explain the selective advantage of GI.1d strains over the strains they have replaced. It would be interesting to search for the presence of genomic RNA in liver tissue samples from infected rabbits before 16 hpi to know more precisely when this target organ of pathogenic lagoviruses was infected. Similarly, a higher frequency of collection points between 24 and 39 hpi would provide information on the order in which the other organs were infected. The minimum dose of infectious GI.1d/00–21 viral particles causing mortality in orally inoculated rabbits is higher than expected, and these data will be useful for subsequent studies requiring its multiplication in vivo. Similarly, it may be interesting to assess viral distribution in rabbits infected with different viral doses to observe their impact on virus replication in vivo.

## Methods

### Origin and molecular characterisation of the GI.1/00–21 strain

#### Sample collection and virus detection

The GI.1d/00–21 sample was collected in 2000 from a rabbit found dead by the French network for epidemiological surveillance of wildlife diseases and poisonings (SAGIR). The sample was screened and detected positive for RHDV by the Inovalys-Angers laboratory (Angers, France). One mL of liver exudate (liquid collected after liver freeze-thawing) was sent to the ANSES laboratory and stored at − 20 °C for research.

#### Genome amplification and sequencing

Total RNA was extracted from 100 μL of liver exudate using an RNeasy Mini kit (Qiagen), according to the manufacturer’s instructions. RNA was reverse transcribed using oligo-dT (Invitrogen) as a primer and SuperScript™ II Reverse Transcriptase (Invitrogen). cDNA was amplified using AmpliTaq Gold DNA polymerase (Applera Applied Biosystems). To sequence the complete coding genome of GI.1d/00–21, four overlapping PCRs were used to amplify cDNA (see primers in Additional file 1). To confirm the sequence obtained, further amplification of cDNA was performed using a pair of primers designed to specifically amplify the GI.1d/00–21 coding genome in a single step (RHD-21Fwd and RHD-7410Rev, see Additional file 1). The genome extremities were acquired using the primers and the rapid amplification of cDNA ends (RACE) method described in Lemaitre et al. [33]. All PCR products were visualised by electrophoresis on agarose gel and were purified using a MinElute™ PCR Purification Kit (Qiagen).

Sequencing was performed in both directions using PCR primers and several inner primers (primer sequences available upon request) and Big Dye Terminator v3.1 (Life Technologies), as recommended by the manufacturer, then analysed with an ABI Prism 3130 Genetic Analyzer (Applied Biosystems). The consensus sequences, then the complete genome sequence, were compiled using Vector NTI Advance 11.5 (Invitrogen). For genotyping, the sequence of the gene encoding the capsid protein (VP60) was aligned against the nucleotide sequences available in databases using the standard nucleotide BLAST (blastn) program from the National Center for Biotechnology Information (NCBI) web BLAST service. For the cleavage site determinations and the gene identifications, the deduced sequence of the ORF1 polyprotein was obtained using EMBOSS Transeq software (EMBL-EBI website), and was compared with a GI.1 polyprotein sequence for which cleavage sites have been experimentally confirmed [7].

#### Phylogenetic and recombination analyses

The 631 GI.1 and GI.2 VP60 gene sequences available in nucleotide databases at the time of analysis were aligned using ClustalW in MEGA software version 7 [34]. Jalview2 software [35] was used to reduce the number of sequences with a redundancy threshold of 98%. The final dataset included 121 VP60 gene sequences. The phylogenetic relationships among the selected lagovirus capsid sequences were estimated using the maximum likelihood (ML) method with MEGA7. This analysis utilised the GTR + Γ model of nucleotide substitution and NNI branch-swapping. Trees were rooted using a GII.1 VP60 sequence (accession number AM933650). The reliability of the consensus trees was assessed by bootstrap with 1000 replicates.

The entire GI.1d/00–21 genome sequence was screened for recombination using seven methods (RDP, GENECONV, BootScan, MaxChi, Chimaera, SiScan and 3Seq) available in RDP software, version 4.97 [36].

### In vivo studies

#### Animals and experimental treatments

In all trials, 10-week-old specific-pathogen-free (SPF) New Zealand White rabbits (Hypharm, Roussay, France, or Charles River Laboratory, Saint-Germain-Nuelles, France) were housed in cages with environmental enrichment (platform, tunnel) under BSL2 biosafety conditions with ad libitum access to water and to commercial rabbit pellet feed throughout the entire experiment. The rabbits were acclimated for a minimum of 3 days before the first experimental manipulations. The rabbits were monitored three times a day for signs of terminal disease after the viral inoculation, indicated by animal isolation, anorexia and little resistance to handling. When these humane endpoints were reached, the rabbit was euthanised. For each euthanasia, the rabbit was stunned by electronarcosis (Assommoir VE Memory, FAF, Saint-Sernin-Sur-Rance, France), followed immediately by the sectioning of both carotids.

#### Virus multiplication and sequence confirmation

The GI.1d/00–21 virus was first propagated in vivo in order to generate a homogeneous viral stock to be used as an inoculum in subsequent experiments. For this purpose, 10 rabbits were housed in the same cage. Of these, one rabbit was inoculated by the intramuscular route with 200 μL of inoculum. The inoculum was composed of 50 μL of GI.1d/00–21-infected liver exudate (the amount of viral material available in the laboratory) diluted in 150 μL PBS and was treated with 10,000 U of penicillin, 10 mg/mL of streptomycin, 0.25 mg/mL of amphotericin B (Fungizone), and 0.25 mg/mL of gentamicin for 1 h15 on ice prior to inoculation. Post-mortem examinations were performed during the trial on rabbits found dead or euthanised after the observation of humane endpoints, as well as on surviving rabbits euthanised at the end of the trial (8 dpi). Blood and liver samples were collected from euthanised rabbits and only liver samples on animals found dead.

To check for the presence of lagovirus in each liver sample, total RNA was extracted from 100 μL of liver exudate using a NucleoSpin® RNA kit (Macherey-Nagel), according to the manufacturer’s instructions. One-step reverse transcriptions and amplifications were performed using primers U38 and L337 that amplify part of the capsid protein gene [37] and SuperScript™ III One-Step Platinum Taq HiFi (Invitrogen). The viral genomes of the PCR-positive samples were sequenced and analysed at the Next-Generation Sequencing platform located at the ANSES laboratory. The liver estimated to contain a large amount of virus based on one-step RT-PCR results and whose viral genomic sequence was identical to the consensus sequence of the wild-type virus was stored at − 20 °C and used for the experimental studies. In addition, sequences of the genomic ends of the amplified virus were acquired as described above to confirm the identity of its complete genomic sequence with that of the wild-type strain.

#### Replication kinetics assay and characterisation of the GI.1d/00–21 phenotype

##### Inoculum preparation and viral titration

The inoculum was composed of 30 mL 2% w/v GI.1d/00–21 liver homogenate in PBS previously homogenised using a mixer-mill disruptor (TissueLyser, Qiagen), then clarified at 10000 *g* for 10 min prior to dilution. Two mL of the inoculum were stored at − 20 °C for later estimation of the genome equivalent after a viral purification step. For this purpose, we used centrifugation through an iodixanol gradient (Optiprep™ solution, Stemcell Technologies) as described in Teixeira et al. [26], except that the gradient with the clarified liver homogenate was centrifuged at 304000 *g* for 1 h 31 min with an SW 55 Ti swinging-bucket rotor in a Beckman ultracentrifuge. RNA extraction and RT-qPCR were performed from the purified fraction. The genome equivalent of the purified virus estimated by RT-qPCR was 10^11^ gRNA copies/mL. Thus, each rabbit was inoculated with a very high infective dose (around 10^11^ genomic RNA copies).

##### In vivo *study of early virus replication kinetics*

Twenty-four rabbits were housed in the same cage in a BSL2 containment cell. Two uninfected control rabbits were housed in the same cage in another containment cell. The 24 rabbits were inoculated each with 1 mL of the inoculum via the oral route. The uninfected control rabbits were inoculated each with 1 mL of PBS.

To determine the replication kinetics of GI.1d/00–21, two rabbits were euthanised at 16, 20, 24, 39, and 45 hpi, respectively. The last point of this study (60 hpi) corresponded to the time when the two first rabbits that died from viral infection were found. The samples collected at necropsy were blood (except for the two rabbits found dead at 60 hpi), thymus, lungs, liver, spleen, kidneys, duodenum, faeces and rectal swab.

##### Characterisation of the GI.1d/00–21 phenotype

In order to evaluate the RHD signs induced by the GI.1d/00–21 strain in vivo, the remaining rabbits were monitored three times a day, or more frequently when required, between 60 hpi and the end of the assay (8 dpi). Post-mortem examinations were performed on all rabbits found dead or euthanised during the experiment and at the end. Blood and liver samples were collected from infected rabbits euthanised when humane endpoints were reached and only liver samples on animals found dead. Blood and the same tissue samples as those of the infected rabbits used for the kinetic assay were collected from one uninfected control rabbit.

#### Viral dose effect assay

##### Inoculum preparation

GI.1d/00–21 infected liver homogenate (15% w/v) was purified through an iodixanol gradient, as described above. RNA from the purified virus was extracted, as described below, and genome equivalent of the purified virus was estimated by RT-qPCR. Serial dilutions were then prepared in PBS and three doses were selected as inoculum: 10^− 1^ (negative control dose), 10^4^ (low dose) and 10^7^ (high dose) gRNA copies per animal.

##### In vivo *estimation of the minimum infective dose*

Three BSL2 containment cells were allocated to infection studies (10 rabbits in a same cage per cell) and a fourth for mock-inoculation (two rabbits in a same cage). Nine of 10 rabbits per cell were inoculated by the oral route with 10^− 1^, 10^4^ or 10^7^ viral RNA copies per rabbit, respectively.

The infection of the non-inoculated in-contact rabbit was used to identify the time post inoculation when horizontal transmission from infected pen-mates, rather than direct infection by the inoculum, could be responsible for infection. When the in-contact rabbit died, the surviving rabbits in the containment cell were euthanised. These rabbits and the in-contact rabbit were not included in calculation of the case fatality rate.

Post-mortem examinations were performed on all rabbits found dead or euthanised during the experiment. Blood and liver samples were collected on euthanised infected rabbits and only liver samples on animals found dead.

#### RNA extraction, viral quantification by quantitative reverse-transcriptase PCR (RT-qPCR) and detection by RT-PCR

RT-qPCR was used to monitor viral RNA load in inocula and in samples collected from inoculated rabbits. To quantify viral RNA in the inocula, 100 μL of GI.1d/00–21 liver homogenate or of purified virus fractions were used for the RNA extraction using a NucleoSpin® RNA kit (Macherey-Nagel), according to the manufacturer’s instructions. To quantify viral RNA in tissue samples collected from rabbits, between 15 and 30 mg of collected tissue were homogenised using a mixer-mill disruptor (TissueLyser, Qiagen), then centrifugated for 30 s at 10000 rpm. For the rectal swabs, each swab was incubated in 1 mL of PBS then vortexed for 30 s and centrifugated for 30 s at 10000 rpm.. Total RNA was extracted from 200 μL of each supernatant using a NucleoMag Vet kit (Macherey-Nagel) in a KingFisher Flex™ (Thermo Fisher Scientific) automated magnetic collection device, according to the manufacturer’s instructions.

A standard range of RNA transcripts for GI.1d/00–21 strain was prepared to evaluate the limit of quantification of the RT-qPCR. In vitro transcription reactions were used to synthesize RNA transcripts from DNA templates using a T7 promoter system. cDNA was amplified with primers T7 + 1317Fwd (5′ TTAATAATACGACTCACTATAGGGGTGGCCAAGGACCTCAC 3′), incorporating a T7 polymerase recognition sequence at the immediate 5′ end, and the reverse primer 1444Rev (5′ AGGTGTTGGTTGTATGATGG 3′) using Phusion Green Hot Start II High Fidelity DNA Polymerase (ThermoFisher Scientific). These primers were designed in the non-structural part of the genome to amplify genomic RNA (gRNA) only. PCR products were visualised by electrophoresis on agarose gel then purified using a MinElute® TM PCR Purification Kit (Qiagen) and quantified by Qubit® Fluorometer (Life Technology). In vitro transcription using 3 μg of DNA template was done at 37 °C for 4 h using RiboMAX™ Large Scale RNA Production Systems (Promega). Products were then treated twice with 7.5 U of DNaseI using the RNase-free DNase Set (Qiagen) to remove template DNA. Purification of RNA transcripts was then performed using an RNeasy® Mini Kit (Qiagen), followed by quantification using a Qubit® Fluorometer. RNA concentrations were converted to copy number using the following formula:

$$ Y\ \left( molecules/\mu L\right)=\left(\frac{\mathrm{X}\ \left(\mathrm{g}/\upmu \mathrm{L}\ \mathrm{RNA}\right)}{\left(\mathrm{transcript}\ \mathrm{length}\ \mathrm{in}\ \mathrm{nucleotides}\right)\times 340}\right)\times 6.023\times {10}^{23} $$

Genomic RNA was amplified and quantified using a Power SYBR® Green RNA-to-CT™ 1-Step kit (Thermo Fisher Scientific) with the primer 1342Fwd (5′ ATACAGCAAAAGGTTATGACAG 3″) and 1444Rev. Serial dilutions of in vitro RNA transcribed were used as standards in RT-qPCR. The detection limit for GI.1d/00–21 was estimated at 100 copies per reaction.

The negative samples in RT-qPCR were analysed again with one-step reverse transcriptions and amplifications performed using U38 and L337 as primers [37] and SuperScript™ III One-Step Platinum Taq HiFi (Invitrogen) in order to confirm or reject results.

#### Serology

Blood samples collected during the in vivo assays were centrifuged at 1500 *g*, 15 min at 4 °C to separate the plasma. Sera were analysed in duplicate for the presence of anti-VP60 RHDV antibodies by the commercial enzyme-linked immunosorbent assay (ELISA) INgezim RHDV 17.RHD.K1 kit (Eurofins-Ingenasa), using the manufacturer’s instructions. This ELISA kit uses a peroxidase-conjugated protein A that can bind to different immunoglobulin isotypes and was described to detect anti-VP60 GI.1 strain [38].

#### Statistics

The time interval to death was investigated with Kaplan–Meier Survival Plots and compared statistically between groups using log-rank tests using R version 3.6.1 in RStudio version 1.1.463 [39].

## Supplementary Information

**Additional file 1.** List of primers used to amplify and/or to sequence the GI.1d/00–21 complete coding sequence.

## Data Availability

The GI.1d/00–21 genome sequence is publicly available in the GenBank database (https://www.ncbi.nlm.nih.gov/genbank) under accession number MH190418. The data that support the findings of this study are included within the published article and its supplementary information file. The other datasets generated during this study including the inner primer sequences used to sequence the genome are available from the corresponding author on request.

## References

[1] Gavier-Widèn D, Mörner T (1991). Epidemiology and diagnosis of the European brown hare syndrome in Scandinavian countries: a review. Rev Sci Tech.

[2] Abrantes J, Van Der Loo W, Le Pendu J, Esteves PJ (2012). Rabbit haemorrhagic disease (RHD) and rabbit haemorrhagic disease virus (RHDV): a review. Vet Res.

[3] Lavazza A, Capucci L (2016). Rabbit Haemorrhagic disease. OIE Terrestrial Manual.

[4] Le Gall-Reculé G, Zwingelstein F, Boucher S, Le Normand B, Plassiart G, Portejoie Y, Decors A, Bertagnoli S, Guérin J-L, Marchandeau S (2011). Detection of a new variant of rabbit haemorrhagic disease virus in France. Vet Rec.

[5] Dalton KP, Nicieza I, Balseiro A, Muguerza MA, Rosell JM, Casais R, Alvarez AL, Parra F (2012). Variant rabbit hemorrhagic disease virus in young rabbits, Spain. Emerg Infect Dis.

[6] Meyers G, Wirblich C, Thiel HJ (1991). Rabbit hemorrhagic disease virus--molecular cloning and nucleotide sequencing of a calicivirus genome. Virology.

[7] Meyers G, Wirblich C, Thiel HJ, Thumfart JO (2000). Rabbit hemorrhagic disease virus: genome organization and polyprotein processing of a calicivirus studied after transient expression of cDNA constructs. Virology.

[8] Abrantes J, Droillard C, Lopes AM, Lemaitre E, Lucas P, Blanchard Y, Marchandeau S, Esteves PJ, Le Gall-Reculé G (2020). Recombination at the emergence of the pathogenic rabbit haemorrhagic disease virus Lagovirus europaeus/GI.2. Sci Rep.

[9] Le Pendu J, Abrantes J, Bertagnoli S, Guitton JS, Le Gall-Reculé G, Lopes AM, Marchandeau S, Alda F, Almeida T, Celio AP, Barcena J, Burmakina G, Blanco E, Calvete C, Cavadini P, Cooke B, Dalton K, Delibes Mateos M, Deptula W, Eden JS, Wang F, Ferreira CC, Ferreira P, Foronda P, Goncalves D, Gavier-Widèn D, Hall R, Hukowska-Szematowicz B, Kerr P, Kovaliski J, Lavazza A, Mahar J, Malogolovkin A, Marques RM, Marques S, Martin-Alonso A, Monterroso P, Moreno S, Mutze G, Neimanis A, Niedzwiedzka-Rystwej P, Peacock D, Parra F, Rocchi M, Rouco C, Ruvoën-Clouet N, Silva E, Silverio D, Strive T, Thompson G, Tokarz-Deptula B, Esteves P (2017). Proposal for a unified classification system and nomenclature of lagoviruses. J Gen Virol.

[10] Le Gall-Reculé G, Zwingelstein F, Laurent S, De Boisseson C, Portejoie Y, Rasschaert D (2003). Phylogenetic analysis of rabbit haemorrhagic disease virus in France between 1993 and 2000, and the characterisation of RHDV antigenic variants. Arch Virol.

[11] Fitzner A, Niedbalski W (2017). Phylogenetic analysis of rabbit haemorrhagic disease virus (RHDV) strains isolated in Poland. Arch Virol.

[12] Abrantes J, Lopes AM, Lemaitre E, Ahola H, Banihashem F, Droillard C, Marchandeau S, Esteves PJ, Neimanis A, Le Gall-Reculé G (2020). Retrospective analysis shows that Most RHDV GI.1 strains circulating since the late 1990s in France and Sweden were recombinant GI.3P-GI.1d strains. Genes.

[13] Mitro S, Krauss H (1993). Rabbit hemorrhagic disease: a review with special reference to its epizootiology. Eur J Epidemiol.

[14] Prieto JM, Fernandez F, Alvarez V, Espi A, Garcia Marin JF, Alvarez M, Martin JM, Parra F (2000). Immunohistochemical localisation of rabbit haemorrhagic disease virus VP-60 antigen in early infection of young and adult rabbits. Res Vet Sci.

[15] Guittré C, Ruvoën-Clouet N, Barraud L, Cherel Y, Baginski I, Prave M, Ganière JP, Trepo C, Cova L (1996). Early stages of rabbit haemorrhagic disease virus infection monitored by polymerase chain reaction. Zentralbl Veterinarmed B.

[16] Shien JH, Shieh HK, Lee LH (2000). Experimental infections of rabbits with rabbit haemorrhagic disease virus monitored by polymerase chain reaction. Res Vet Sci.

[17] Alonso C, Oviedo JM, Martin-Alonso JM, Diaz E, Boga JA, Parra F (1998). Programmed cell death in the pathogenesis of rabbit hemorrhagic disease. Arch Virol.

[18] Forrester NL, Moss SR, Turner SL, Schirrmeier H, Gould EA (2008). Recombination in rabbit haemorrhagic disease virus: possible impact on evolution and epidemiology. Virology.

[19] Lopes AM, Magalhães MJ, Alves PC, Esteves PJ, Abrantes J (2017). An update on the rabbit hemorrhagic disease virus (RHDV) strains circulating in Portugal in the 1990s: earliest detection of G3-G5 and G6. Arch Virol.

[20] Le Gall G, Huguet S, Vende P, Vautherot JF, Rasschaert D (1996). European brown hare syndrome virus: molecular cloning and sequencing of the genome. J Gen Virol.

[21] Lopes AM, Gavier-Widèn D, Le Gall-Reculé G, Esteves PJ, Abrantes J (2013). Complete coding sequences of European brown hare syndrome virus (EBHSV) strains isolated in 1982 in Sweden. Arch Virol.

[22] Droillard C, Lemaitre E, Chatel M, Guitton JS, Marchandeau S, Eterradossi N, Le Gall-Reculé G (2018). First complete genome sequence of a hare Calicivirus strain isolated from Lepus europaeus. Microbiol Res Announc.

[23] Alhatlani B, Vashist S, Goodfellow I (2015). Functions of the 5′ and 3′ ends of calicivirus genomes. Virus Res.

[24] Smith DR, Aguilar PV, Coffrey LL, Gromowski GD, Wang E, Weaver SC (2006). Venezuelan equine encephalitis virus transmission and effetc on pathogenesis. Emerg Infect Dis.

[25] Teunis PFM, Moe CL, Liu P, Miller SE, Lindesmith L, Baric RS, Le Pendu J, Calderon RL (2008). Norwalk virus: how infectious is it?. J Med Virol.

[26] Teixeira L, Marques RM, Aguas AP, Ferreira PG (2011). A simple and rapid method for isolation of caliciviruses from liver of infected rabbits. Res Vet Sci.

[27] Gall A, Hoffmann B, Teifke JP, Lange B, Schirrmeier H (2007). Persistence of viral RNA in rabbits which overcome an experimental RHDV infection detected by a highly sensitive multiplex real-time RT-PCR. Vet Microbiol.

[28] Duarte MD, Carvalho CL, Barros SC, Henriques AM, Ramos F, Fagulha T, Luís T, Duarte EL, Fevereiro M (2015). A real time Taqman RT-PCR for the detection of rabbit hemorrhagic disease virus 2 (RHDV2). J Virol Methods.

[29] Liu W, Dang R, Wang X (2015). Development of a SYBR-based real-time PCR to detect rabbit hemorrhagic disease virus (RHDV) and analyze its tissue distribution in experimentally infected rabbits. Virol Sin.

[30] Plassiart G, Guelfi JF, Ganière JP, Wang B, André-Fontaine G, Wyers M (1992). Hematological parameters and visceral lesions relationships in rabbit viral hemorrhagic disease. Zentralbl Veterinarmed B.

[31] Yezli S, Otter JA (2011). Minimum infective dose of the major human respiratory and enteric viruses transmitted through food and the environment. Food Environ Virol.

[32] Cooke BD, Berman D (2000). Effect of inoculation route and ambient temperature on the survival time fo rabbits, Oryctolagus cuniculus (L.), infected with rabbit haemorrhagic disease virus. Wildl Res.

[33] Lemaitre E, Zwingelstein F, Marchandeau S, Le Gall-Reculé G (2018). First complete genome sequence of a European non-pathogenic rabbit calicivirus (lagovirus GI.3). Arch Virol.

[34] Kumar S, Stecher G, Tamura K (2016). MEGA7: molecular evolutionary genetics analysis version 7.0 for bigger datasets. Mol Biol Evol.

[35] Waterhouse AM, Procter JB, Martin DMA, Clamp M, Barton GJ (2009). Jalview version 2—a multiple sequence alignment editor and analysis workbench. Bioinformatics.

[36] Martin DP, Murrell B, Golden M, Khoosal A, Muhire B (2015). RDP4: detection and analysis of recombination patterns in virus genomes. Virus Evol.

[37] Le Gall-Reculé G, Zwingelstein F, Fages MP, Bertagnoli S, Gelfi J, Aubineau J, Roobrouck A, Botti G, Lavazza A, Marchandeau S (2011). Characterisation of a non-pathogenic and non-protective infectious rabbit lagovirus related to RHDV. Virology.

[38] Gil F, Titarenko E, Terrada E, Arcalis E, Escribano JM (2006). Successful oral prime-immunization with VP60 from rabbit haemorrhagic disease virus produced in transgenic plants using different fusion strategies. Plant Biotechnol J.

[39] RStudio Team (2019). RStudio: integrated development for R.

