# Supplementary material for: Rabbit haemorrhagic disease virus Lagovirus europaeus/GI.1d strain: genome sequencing, in vivo virus replication kinetics, and viral dose effect
Source: BMC Vet Res. 2021 Jul 28;17:257. doi: 10.1186/s12917-021-02962-2 (PMC8320185; doi:10.1186/s12917-021-02962-2)
Supplement: Supplementary file 1 — Additional file 1. List of primers used to amplify and/or to sequence the GI.1d/00–21 complete coding sequence. [file 12917_2021_2962_MOESM1_ESM.docx]

| Primer name | Primer sequence (5'-3') | Position (relative to GenBank accession number MH190418) (bp) |
| --- | --- | --- |
|  |  |  |
| 1U | GATTAGGCCGTGAAAGTTATG | 1-13 |
| 1L | CAACGTCAACAAACTTGTCC | 552–5 32 |
| 7L | CCACTGCCGTTGTGTCTCAT | 3369-3350 |
| 13L | GCACGAACGACATGTCAGG | 6332-6314 |
| 15L | ATCAAGCACTGGACTCGCC | 7378-7362 |
| RHD-3082Fwd | CATGACATGACTGCAGAGGAG | 3092-3112 |
| RHD-5461Rev | GCCAATCCCTGCCGTTGCAATCG | 5473-5450 |
| RHD-21Fwd | GTGAAAGCTATGGCGGTTATG | 1-21 |
| RHD-7410Rev | ATAGCTTGCTTTAAACTATAAACCCAATT | 7437-7409 |
